# “Exercise during hemodialysis and health promoting behaviors: a clinical trial”

**DOI:** 10.1186/s12882-019-1276-3

**Published:** 2019-03-19

**Authors:** Alireza Dashtidehkordi, Nahid Shahgholian, Fatemeh Attari

**Affiliations:** 1 Department of Dialysis, Alzahra Hospital, Isfahan University of Medical Sciences, Isfahan, Iran; 2 Kidney Diseases Research Center, Department of Critical Care Nursing, School of Nursing and Midwifery, Isfahan University of Medical Sciences, Isfahan, Iran; 3 MS of Critical Care Nursing, Amin Hospital, Isfahan University of Medical Sciences, Isfahan, Iran

**Keywords:** Exercise, Hemodialysis, Health promotion

## Abstract

**Background:**

Health promoting behaviors are among the determinants of health. Hemodialysis causes significant changes in the lives of patients and affects their health promoting behaviors. Accordingly, this study aimed at investigating the effect of exercise during hemodialysis on health promotion behaviors in patients undergoing hemodialysis.

**Methods:**

This study was a two-stage (before and after) clinical trial. The sample of the study consisted of 60 hemodialysis patients in two hospitals in Isfahan who were selected randomly and divided into two groups of control and intervention using random allocation method. A 8-week exercise program by stationary bicycles (Mini-bike) was designed for the intervention group, while the control group underwent a 10-min limbering exercise for 8 weeks. Data were collected using demographic questionnaire and the Health Promoting Lifestyle Profile II (HPLP-II) questionnaire before and after the intervention and were analyzed using SPSS21 software.

**Results:**

Based on the independent t-test results, no significant difference was observed between the mean score of health promoting behaviors and its areas before the intervention (*P* > 0.05). However, the results of this test showed that the mean score of health promoting behaviors and its areas, except for the areas of responsibility (*P* = 0.052) and spirituality (*P* = 0.211), was significantly different between the two groups after the intervention (*p* < 0.05).

**Conclusions:**

The results of this study showed that exercise with stationary bicycle during hemodialysis could promote health promoting behaviors. Thus, this exercise is recommended to be considered as part of the therapeutic protocol of these patients in hemodialysis departments.

**Trial registration:**

The clinical trial was found to be in accordance to the ethical principles and the national norms and standards for conducting medical research in Iran. IRCT registration number: IRCT20150116020675N3. Registration date: 2019-01-18, 1397/10/28

**Approval ID:** IR.MUI.RESEARCH.REC.1397.014

**Approval Date:** 2018-07-01

**Evaluated by:** Vice-Chancellor in Research Affairs -Medical University of Isfahan

## Background

Hemodialysis is considered to be one of the most commonly used alternative treatments for renal function [1]. According to statistics, 15% is added annually to the number of hemodialysis patients in Iran; as such, this figure will reach to 3,500,000 by 2020 that will be 7% higher than the global rate [2]. Although hemodialysis increases the life span of patients with renal insufficiency, it can effect remarkable changes such as reduced efficiency and ability to perform activities, social isolation, immobility, reduced self-confidence and, finally, disappointment at the future and continuation of the treatment. In addition to encountering many physiological changes, these patients face many mental and psychological stresses each of which in turn can disturb their mental status and sleep quality. As such, most of these patients are not adapted to problems and tensions, and develop disorders such as anxiety, depression and isolation which affect their health status and weaken their health promoting behaviors over time [3–6]. This stress of the patients can also affect their nurses and make them stressful [7].

Health promoting behaviors refer to those behaviors that make people able to improve their own and their society’s health. The areas of health promoting behaviors include nutrition, physical activity, stress management, health responsibilities, interpersonal relationships, and spiritual growth [8, 9]. These behaviors are important as they have the potentiality to prevent the complications of illness and treatment, reduce pathogenicity, improve quality of life, maintain the function and independence of individuals, and reduce the burden of care on society. These behaviors are considered to be important determinants of health, and promoting them can prevent one third of deaths and the risk of heart disease in these patients [10, 11].

Several studies have shown that health promoting behaviors in patients undergoing hemodialysis are affected by renal disease and hemodialysis treatment. For example, studies conducted by Ma et al. (2013) demonstrated that health promoting behaviors in hemodialysis patients are not in a desirable level and some interventions are needed to improve these behaviors [12]. Today, promotion of these behaviors is one of the main challenges faced by health care providers, including nurses [13]. Exercise is one of the interventions that can improve health promotion behaviors [14]. Johansen (2007) indicated that exercise improves the health behaviors of patients with end-stage renal failure [15]. Capitanini et al. (2008) also showed that exercise can contribute to the improvement of physical performance, promotion of public health and quality of life in hemodialysis patients [16]. On the other hand, some studies have shown that exercise is safe during dialysis and does not cause physical damage and does not interfere with the hemodialysis process [17]. Similar studies have proven the positive effect of exercise during dialysis on the physical symptoms of patients; but the effect of this method on promoting other aspects of health has been less investigated [18]. Accordingly, as care for these patients in addition to the physical dimension should include other dimensions of health [19] and as there is a need for a holistic approach, this study aimed at determining the effect of exercise during dialysis on health promoting behaviors in hemodialysis patients.

## Methods

This study was a two-group, two-stage clinical trial that, approved by the Ethics Committee of Isfahan University of Medical Sciences, was conducted in Iran. This study was conducted in the Noor and Al-Zahra hospitals, Isfahan, with the participation of 60 hemodialysis patients who were eligible to be included in the study. Sampling was performed randomly and in the first meeting the purpose of the research was explained and informed consent was obtained from the patients. Then, using random allocation method, the samples were divided (in closed packets) into two groups of intervention (*n* = 30) and control (n = 30).

### Inclusion criteria

Aged between 18 and 65, a history of at least 3 months of hemodialysis [20], no physical and mental disability, no known ischemic heart disease, no myocardial infarction (heart attack) and angina during the last 3 months, based on the patients’ history [17], no acute pulmonary disease so that the patient needs oxygen therapy during dialysis [21], no history of stroke or transient ischemic attacks over the past 3 months [22], no skeletal-muscle disorder that prevent the patient from exercising (pedaling the stationary bicycle) [17], and doing three sessions of 4-h dialysis during the week.

### Exclusion criteria

Unwillingness to continue participating in the study, the presence of any disorder, including cardiovascular, pulmonary and musculoskeletal disorders during the study which may prevent the patient from exercise, and not doing the exercises for three consecutive sessions and six non-consecutive sessions [17].

### Data collection

Two questionnaires were used as the data collection tool. The first questionnaire was related to demographic information including age, sex, occupation, underlying diseases and duration of hemodialysis treatment. The second questionnaire, related to the Health Promoting Lifestyle Profile II (HPLP-II), included 52 questions in 6 sub-scales of nutrition (9 questions, scored 9 to 36), physical activity (8 questions, scored 8 to 32), stress management (8 questions, scored 8 to 32), interpersonal relationships (9 questions, scored 9 to 36), health-related responsibility (9 questions, scored 9 to 36), and spiritual growth (9 questions, scored 9 to 36). Sixty min was given to each person to complete the questionnaire. When filling the questionnaire, the patient could rest. Based on a 4-point Likert Scale (never, sometimes, often, and usually), the questionnaire asked the respondents to determine that to what extent they have the health promoting behaviors. In general, the scores of both the Health Promoting Lifestyle Profile II and behavioral area were calculated for the all 52 questions and each sub-scale (8–9 items) using the average of responses. The total score of health promoting behaviors ranged from 52 to 208, and for each area a separate score could be calculated that a higher score suggested having more health promoting behaviors [22].

Data were collected in this study using HPLP-II questionnaire. HPLP-II is the modified version of HPLP developed by Walker et al. to measure health promoting behaviors, focusing on inventive tasks and individual perceptions whose function is to maintain or increase levels of well-being, self-actualization, and individual satisfaction. Walker and Hill-Polerecky considered the Cronbach’s Alpha of 0.94 for HPLP-II and reported a range of 0.79 to 0.94 for its six sub-scales [23].Mohammadi Zeidi et al. (2012) also used the data collected from a cross-sectional study as well as exploratory and confirmatory factor analysis for the structural validity of the questionnaire. Retest method with a two-week interval was used for determining the reliability of the tool, and the Cronbach’s alpha coefficient for examining its internal consistency. Based on the results of the study, the Cronbach’s alpha coefficient for the whole tool was reported to be 0.82 and for the sub-scales ranged from 0.44 to 0.91. As such, the findings showed that the Persian version of the health promoting behaviors questionnaire has acceptable validity and reliability [22].

#### Intervention

An exercise program was performed for the patients of the intervention group; so that, 30 min after the initiation of dialysis and during the first 2 h of it, the patients exercised for two half hours with 5-min intervals using a stationary bicycle (Mini-bike made in Taiwan).The intensity of the exercise was determined by the patient based on the rotational speed of the bike. The machines minimum rotational speed was set at 15 rpm but the patient could increase it based on his/her ability and tolerance. This exercise program was conducted for 8 weeks, 3 times a week (every other day). If the patient had a blood pressure of 180/110 mmHg and higher or systolic pressure lower than 90 mmHg or chest pain and shortness of breath before or during dialysis, the exercise was stopped that session. None of the patients had this problem and all of them could continue their exercise without interruption. The patients controlled during the intervention and there was no evidence of inability to continue exercise at any of the sessions.

Blood pressure of the patients was measured before the start of the exercise program and at the rest intervals between the two halves of the exercise program. A program, including 10 stretching exercises (any exercises for 30 s and 1 min rest between them), was performed for the patients of the control group. The health promoting behaviors questionnaire was completed both before the intervention and at the end of the eighth week for all samples of the two groups.

### Statistical tests

For data analysis, SPSS version19 (IBM Corp. Released 2010.IBM SPSS Statistics for Windows, Version 19.0) was used. For qualitative variables such as sex, occupational status and underlying diseases the Chi-square test was used, while for quantitative variables of age and duration of treatment with dialysis, independent t-test was used (Table 1). Paired t-test was used to compare the means in each group, and independent t-test to compare the means between the two groups (Table 2). Significance level was considered to be *P* ≤ 0.05.

**Table 1:** Comparison of demographic variables between the two groups

Variables		Control group		Intervention group		*P* value
		Number	Percent	Number	Percent	
Sex	Female	22	88	24	88.9	0.628
	Man	3	12	3	11.1	
Employment status	Employed	8	32	10	37	0.776
	Unemployed& Retired	17	68	17	63	
Other diseases	To have	22	88	25	92.6	0.662
	Not to have	3	12	2	7.4	
Age	Average age (Year)	55.64		51.22		0.20
Duration of treatment with dialysis	Average years of treatment with dialysis	4.48		5.48		0.16

**Table 2:** Comparison of general health and their domains before and after the intervention in the intervention and control groups

Domains	Times	Study group		Control group		T	*P* value
		Mean	SD	Mean	SD		
Nutrition	Before intervention	19.04	4.26	17.78	6.19	0.79	0.433
	After intervention	22.26	4.311	18.96	5.200	2.498	0.016
	*P* value	0.0		*P* = 0.198		–	–
	*t*	4.058		1.323			
Physical	Before intervention	13.78	3.203	13.36	3.39	0.457	0.65
	After intervention	16.85	4.470	13.72	2.509	3.081	0.003
	*P* value	0.0		0.361		–	–
	*t*	4.053		0.931			
Stress management	Before intervention	13.78	3.203	13.36	3.39	0.457	0.846
	After intervention	16.85	4.470	13.72	2.509	3.081	0.148
	*P* value	0.044		0.4 21 T			–
	*t*	2.102		0.820			
Interpersonal relationship	Before intervention	19.85	4.662	19.32	4.553	0.418	0.678
	After intervention	21.85	4.204	19.40	4.113	2.123	0.039
	*P* value	0.014		0.867		–	–
	*t*	2.643		0.169			
Responsibility	Before intervention	19.63	4.683	19.44	4.134	0.154	0.878
	After intervention	21.44	3.714	19.20	3.764	2.163	0.035
	*P* value	0.052		0.649			
	*t*	2.035		0.461			
Spritual	Before intervention	22.22	4.710	22.40	4.983	0.132	0.895
	After intervention	23.41	2.832	21.56	3.969	1.943	0.058
	*P* value	0.211		0.254			
	*t*	1.282		1.170			
Health promotion behaviors	Before intervention	110.19	18.138	107.76	22.424	0.430	0.669
	After intervention	122.70	15.462	108.56	18.956	2.958	0.005
	*P* value	0.002		0.646			
	*t*	3.427		0.464			

## Results

**Fig. 1:**
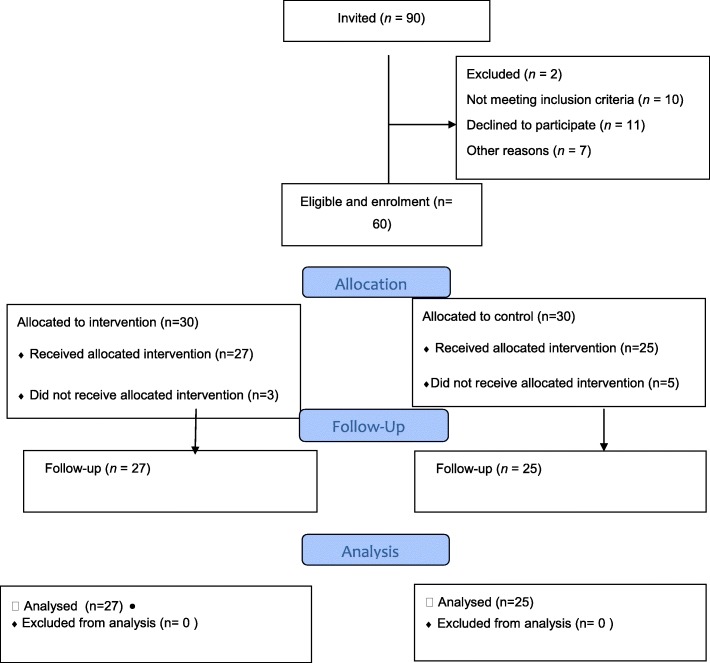
Participant flow and follow up

This study was conducted on 60 hemodialysis patients and, finally, 27 patients of the intervention group and 25 patients of the control group completed the process (Fig. 1).In the intervention and control groups, respectively, 3 and 5 patients get out of the study because did not consent to continue participating in the study. The results of independent t-test showed that there was no significant difference between the two groups in terms of age and duration of hemodialysis (*P* < 0.05). Chi-square test also indicated that there was no significant difference between the two groups in terms of the variables of sex, education level, occupational status and underlying diseases (*p* < 0.05) (Table 1).

Based on the results of independent t-test, it was observed that the mean score of health promoting behaviors (*P* = 0.669) and its areas was not significantly different between the two groups before the intervention (*P* > 0.05). However, according to the results, the mean score of health promoting behaviors (*P* = 0.005) and its areas, except for the areas of responsibility (*P* = 0.052) and spirituality (*P* = 0.211), was significantly different between the two groups after the intervention (*p* < 0.05). According to the results of paired t-test, the mean score of health promoting behaviors was significantly different before and after the intervention (P = 0.005).This difference, however, was not significant in the control group (*P* = 0.669) (Table 2). Table 3 also shows the comparison of average difference scores of general health and their domains before and after the intervention in the control and intervention groups.

**Table 3:** Comparison of average difference scores of general health and their domains before and after the intervention in both groups

Domains	Control group		Intervention group			
	Mean	SD	Mean	SD	t	*P*
Nutrition	1.18	5/0	3.22	0.4	3.1	0.003
Physical	0.36	5/0	3.07	1.4	2.9	0.005
Stress management	0.36	3/0	1.37	0.6	2.9	0.2
Interpersonal relationship	.080	3/0	2	0.9	3	0.004
Responsibility	−0.24	6/0	1.81	1.4	2.5	0.02
Spiritual	−0.84	5/0	1.19	2.1	0.9	0.057
Health promotion behaviors	0.8	7/1	12.51	1.9	4.8	*P* < 0.001

## Discussion

The results related to the evaluation of the effect of exercise on health promoting behaviors showed that exercise can be effective in improving health promoting behaviors except the areas of spirituality and responsibility. Exercise, by using stationary bicycle, could effectively improve the nutritional behaviors of the patients in the intervention group, but stretching exercises had no significant effect on the patients of the control group. Soh et al. (2018) also found out that physical exercises could have a positive impact on the nutritional behaviors in nursing students [24]. Chang et al. (2015), however, showed that Buerger exercises have had no effect on improving nutrition-related behaviors; these authors, however, mentioned that their study was a pilot study with a small sample size and no control group [25].

Physical activity is another area of health promoting behaviors. In this regard, the results of the present study showed an increase in the physical activity of the patients in the intervention group compared with those in the control group. These results are in line with the results obtained by Aucella et al. (2015) who showed that regular physical activity improves the physical function of pre-dialysis patients even in the short term [26]. The results are also in line with the results of the study conducted by Mustata et al. (2011), where they showed that exercise during dialysis can improve physical performance and help people to return from an inactive lifestyle to an active one they have had before the disease [27]. However, the results of the present study are not in line with the results of Logghe I.H et al. (2010), investigating the impact of tai chi exercises on preventing falls and improving physical balance in people over 50 years of age [28]. Among the causes of this difference mention may be made of the difference in the duration of the exercise (1 h twice a week compared to 1 h three times a week), different exercises (tai chi vs. Mini-bike), different measurements of physical function, and different sample size.

Another result of the present study is the effect of exercise on stress management in the intervention group compared to the control group. This result is consistent with the results of Salesi et al. (2014) who investigated the effect of exercise on stress, anxiety, depression and blood pressure of the patients after kidney transplant [29]. Western et al. (2013) believe that exercise, through releasing endorphins and serotonin, and reducing blood cortisol levels, can reduce stress and anxiety and increase feelings of pleasure [30].

The results of the present study also showed that this exercise program could not promote responsibility-related behaviors. Chang et al. (2015) also found that Buerger exercises could not affect responsibility-related behaviors in patients at risk for diabetic foot ulcers. The effect of this exercise program on interpersonal relationships in the intervention group was another result of the present study [25]. The study by Rakhshani et al. (2010) also showed that yoga exercise can have effect on the patients’ interpersonal relationships [31].

In the present study, no evidence was found suggesting the effect of exercise on the spiritual growth of the patient in the intervention group compared with the control group. One of the possible reasons for such a result is the desirable spiritual state of the patient before the beginning of the study in both groups. Lotzke et al. (2016) also compared the effect of yoga and usual exercise in patients with breast cancer and showed that there was no significant difference between the control and the intervention group in terms of spirituality [32]. By contrast, Moadel et al. (2007) showed that yoga exercise was effective on the spiritual dimension of the patients with breast cancer [33].The reason for this difference is maybe the use of different intervention methods. Among the limitations of the study was small sample size, that is, at the time of sampling a small number of patients were eligible to enter the study and this, in turn, reduced the generalizability of the findings of the study. Lack of Mini-bike device in the research environment for intervention was another limitation of the study. In the present study, the intensity of exercise for each patient was not measured. Therefore, it is suggested that future researchers consider this element too.

## Conclusions

Based on the results of this study, showing the effect of exercise with mini-bike during hemodialysis on improving the health promoting behaviors in these patients, it is suggested that measures be taken to ensure that exercise with mini-bike is performed routinely during hemodialysis in hemodialysis departments for eligible patients.

## Abbreviations

HPLP-II: Health Promoting Lifestyle Profile II
MS: Master of Science
N: Number
SD: Standard Deviation
